# Editorial

**Published:** 1912-06

**Authors:** 


### EDITORIAL

The Business Office of the *Journal-Record* is 1 1-2 to 5 1-2 South Broad Street.
The Editorial Office is 1014-15 Century Building.
Address all Business Communications to *Journal-Record of Medicine*, 1 1-2 to 5 1-2 South Broad Street.
Make remittances payable *to The Journal-Record of Medicine*.
On matters pertaining to the Editorial and Original Communications, address Edgar G. Ballenger, M. D., Atlanta, Ga.
Reprints of Original Articles will be furnished at cost price. Requests for the same should always be made in THE MANUSCRIPT.
We will present, postpaid, on request, to each contributor of an original article, twenty (20) marked copies of *The Journal-Record of Medicine* containing such article.

---

The idea of certified milk is not a new one; no further proof of its efficacy in preventing and combating disease is required. All the experiments and demonstrations have been made elsewhere. It now remains for the people of the South to take up the subject and gain the incalculable benefits it offers to the children. Every physician should thoroughly familiarize himself with the subject and use his whole influence in educating the public to recognize the necessity of using healthful milk. The change in infant mortality will more than compensate the people for the slight increase in expense and the better foundations of healthful, normal life given to the children will outlast the generation.

---

CHATTAHOOCHEE VALLEY MEDICAL ASSOCIATION.

We wish to remind our professional brethren of the approaching meeting of the Chattahoochee Valley Medical and Surgical Association, which will be held in Atlanta July 16th and 17th next.

Dr. Hansell Crenshaw, the chairman of the program committee, assisted by our able and indefatigable secretary, Dr. Love, has arranged a scientific program at which the various statewide associations might well sit up and take notice.

The essayists, and those who will join in the discussions, include names known to all—names whose participation mean that we will enjoy a feast of reason and flow of the soul.

The Fulton County Medical Society, as a body, will do the entertaining, and they do say that those in charge have obtained some special pointers from both Epicurus and Lucullus, who are sojourning for the summer in their houseboat on the Styx.

We anticipate a full attendance and a delightful session, so we assure those who come that they will never regret their visit to Atlanta on this auspicious occasion.

G. M. N.

### FEE SPLITTING.

The American Medical Association in its session at Atlantic City has modified the Code of Ethics in attempting to bring it into closer touch with the practical, honorable conduct of the everyday practitioner, and one statement of the law as it now stands takes cognizance of the difficulty the general practitioner often has in collecting what is due to him when he has referred the patient to a specialist. The section is as follows:

"It is detrimental to the public good and degrading to the profession, and, therefore, unprofessional, to give or to receive a commission or to divide a fee for medical advice or surgical treatment, unless the patient or his next friend is fully informed as to the terms of the transaction. The patient should be made to realize that a proper fee should be paid the family physician for the service he renders in determining the surgical or medical treatment suited to the condition, and in advising concerning those best gualified to render any special service that may be required by the patient."

The question arises, "How is the patient to be made to realize that a proper fee should be paid to the physician?" Physiians are frequently called in to find a man suffering with an acute surgical condition, appendicitis for example. The patients are given attention requiring quite as much skill and knowledge as the surgeon subsequently exercises and corresponding benefits are given, but the striking thing is the operation and the matter foremost in the minds of the patient and his family is the operation. No question of the payment to the physician can well be raised at such a time. There is urgent need of action to save life and the doctor must start the processes into activity. It is true that the fee of the surgeon is decided upon, as a rule, and even advance payments may be made. When it is all over and the doctor sends his bill, the answer often enough is, "You did nothing, we have paid the man who did the work." Or else there is long delay and discontent as to the settlement. If the surgeon were to incorporate into his bill an amount commensurate with the physician's services and pay it to him without any suggestion of a rebate, this difficulty would be avoided. We realize that this may open the way to less commendable practice, but no law can keep a man honorable nor make a good man dishonorable. An alternative might be in the insistance on the part of the surgeon that the physician's services should be recognized before the work has commenced, but this is as open to the objection of "commercialism" as anything else. Rules and codes of rules are of little value after all. We know what honesty is and the use of common sense in its practice will keep us close to good conduct. Add "business sense" and the professional man will be able to strike a just balance between the patients' needs and his own requirements in making a living. Sometimes the idea of humanitarian service carries its devotees too far and proves fatal to the very men who would do the good work. That is a sacrifice that is detrimental to the race.

